# JAK inhibitors for the treatment of myeloproliferative neoplasms and other disorders

**DOI:** 10.12688/f1000research.13167.1

**Published:** 2018-01-17

**Authors:** William Vainchenker, Emilie Leroy, Laure Gilles, Caroline Marty, Isabelle Plo, Stefan N. Constantinescu

**Affiliations:** 1 INSERM UMR 1170, Gustave Roussy, Villejuif, France; 2 Université Paris-Saclay, UMR1170, Gustave Roussy, Villejuif, France; 3 UMR 1170, Gustave Roussy, Villejuif, France; 4 Signal Transduction & Molecular Hematology Unit, Ludwig Institute for Cancer Research, Brussels, Belgium; 5 de Duve Institute, Université catholique de Louvain, Brussels, Belgium; 6 Institut National de la Transfusion Sanguine, Paris, France

**Keywords:** JAK inhibitors, MPN, auto-immune diseases, inflammation, allosteric inhibitor

## Abstract

JAK inhibitors have been developed following the discovery of the *JAK2*V617F in 2005 as the driver mutation of the majority of non- *BCR-ABL1 *myeloproliferative neoplasms (MPNs). Subsequently, the search for JAK2 inhibitors continued with the discovery that the other driver mutations ( *CALR* and *MPL*) also exhibited persistent JAK2 activation. Several type I ATP-competitive JAK inhibitors with different specificities were assessed in clinical trials and exhibited minimal hematologic toxicity. Interestingly, these JAK inhibitors display potent anti-inflammatory activity. Thus, JAK inhibitors targeting preferentially JAK1 and JAK3 have been developed to treat inflammation, autoimmune diseases, and graft-versus-host disease. Ten years after the beginning of clinical trials, only two drugs have been approved by the US Food and Drug Administration: one JAK2/JAK1 inhibitor (ruxolitinib) in intermediate-2 and high-risk myelofibrosis and hydroxyurea-resistant or -intolerant polycythemia vera and one JAK1/JAK3 inhibitor (tofacitinib) in methotrexate-resistant rheumatoid arthritis. The non-approved compounds exhibited many off-target effects leading to neurological and gastrointestinal toxicities, as seen in clinical trials for MPNs. Ruxolitinib is a well-tolerated drug with mostly anti-inflammatory properties. Despite a weak effect on the cause of the disease itself in MPNs, it improves the clinical state of patients and increases survival in myelofibrosis. This limited effect is related to the fact that ruxolitinib, like the other type I JAK2 inhibitors, inhibits equally mutated and wild-type JAK2 (JAK2WT) and also the JAK2 oncogenic activation. Thus, other approaches need to be developed and could be based on either (1) the development of new inhibitors specifically targeting *JAK2*V617F or (2) the combination of the actual JAK2 inhibitors with other therapies, in particular with molecules targeting pathways downstream of JAK2 activation or the stability of JAK2 molecule. In contrast, the strong anti-inflammatory effects of the JAK inhibitors appear as a very promising therapeutic approach for many inflammatory and auto-immune diseases.

## Introduction

**Figure 1.**
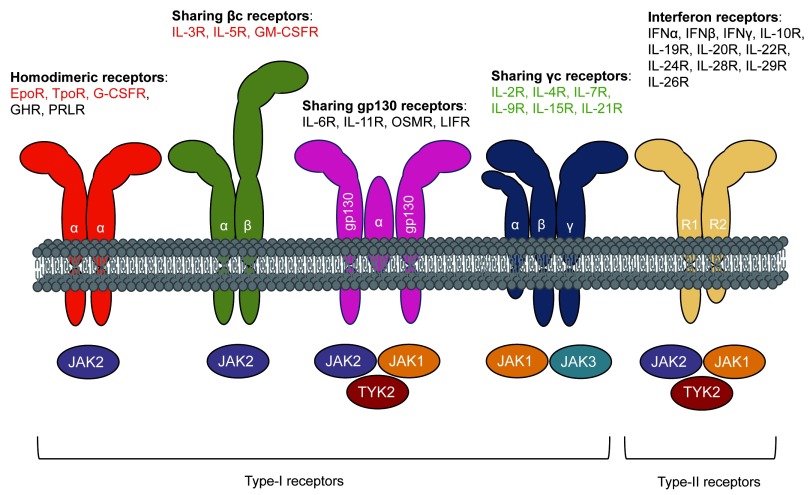
Janus kinases (JAKs) and the cytokine receptor superfamily. Schematic representation of type I and type II cytokine receptor subfamilies based on the extracellular domain sequence homologies. The different JAKs (JAK1, JAK2, JAK3, and TYK2) are employed by each class of receptors, as indicated. Type I receptors can form homodimers (α/α), heterodimers (α/β), or oligomers (gp130/α/gp130);(α/β/γ), although the α chain is mainly responsible for cytokine binding. Cytokine receptor complexes composed of two or more different chains activate at least two different JAKs, while single-chain receptors such as homodimeric receptors activate JAK2 only (although TpoR/MPL and G-CSFR/CSF3R can also use TYK2 and JAK1, respectively). The myelopoiesis-related cytokine receptors are denoted in red, and the lymphopoiesis-related cytokines receptors are denoted in green.

**Figure 2.**
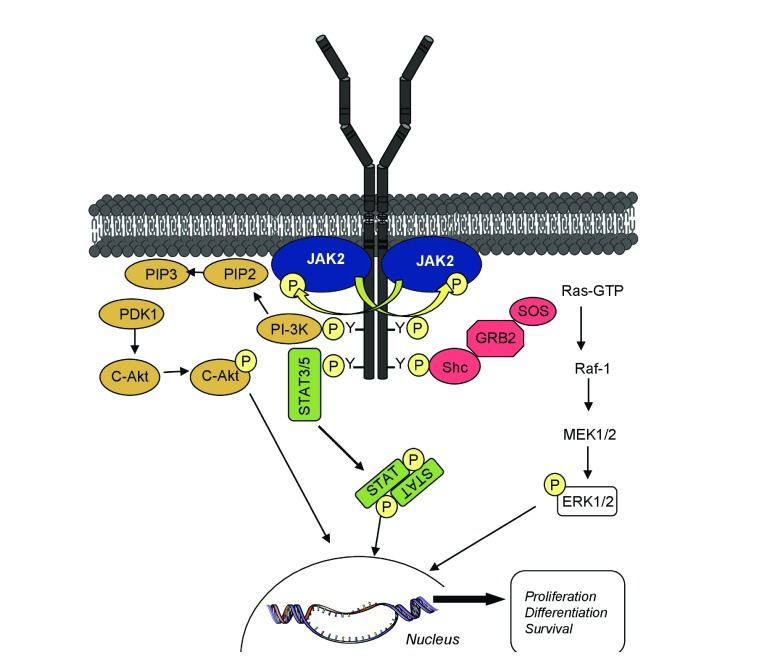
Principal signaling pathways activated by homodimeric cytokine receptors. Cytokine binding to the extracellular domain of receptors induces conformation changes that enable cross-phosphorylation of the appended Janus kinases (JAKs), which then can activate each other. As a result, JAK molecules phosphorylate tyrosine residues on the intracellular part of the receptor, which then can serve as docking sites for SH2 domain containing signaling molecules such as signal transducer and activator of transcription (STAT) but also proteins from the phosphatidylinositol-3′-kinase (PI3K) and mitogen-activated protein kinase (MAPK) pathways.

Janus kinases (JAKs) play a central role in the regulation of hematopoiesis as being mandatory for signaling by receptors for hematopoietic/immunological cytokines ^1^. They control survival, proliferation, and differentiation of hematopoietic cells as well as the function of mature cells by binding to hematopoietic type I and type II cytokine receptors, which are devoid of catalytic activity ( Figure 1). JAKs pre-associated to these receptors to form a functional signaling complex ^1^. Cytokine binding induces or re-orients receptor dimerization, such that JAK kinase domains face each other in a productive conformation for transactivation and phosphorylation of the cytokine receptor cytoplasmic tails ^1^. The latter and JAKs themselves become scaffolds for signaling molecules, particularly for the members of the signal transducer and activator of transcription (STAT) family, which in turn are phosphorylated and homo/hetero-dimerize before translocating to the nucleus. JAK activation also initiates activation of mitogen-activated protein kinase (MAPK), phosphatidylinositol-3′-kinase (PI3K), and AKT/mammalian target of rapamycin (mTOR) ( Figure 2) ^2^.

The JAK/STAT pathway is frequently dysregulated in malignant diseases and in disorders with an abnormal immunological response ^3^.

The discovery that classic *BCR-ABL1*–negative myeloproliferative neoplasms (MPNs) are constantly associated with abnormal JAK2 activation due to different mutations, has paved the way for the development of JAK inhibitors in the therapy of these disorders as well as of other diseases with either genetic alterations in the JAK pathway or JAK-induced activation by autocrine and paracrine cytokine loops ^4, 5^. Here, we focus on the role of JAKs as potential therapeutic targets, the development of JAK inhibitors and their limitations, and potential new strategies targeting the JAKs.

## Janus kinases

**Figure 3.**
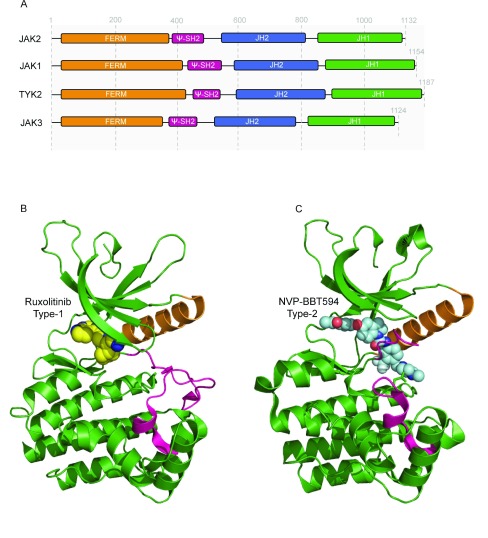
The kinase domain of Janus kinase 2 (JAK2) has been commonly targeted by the current inhibitors. ( **A**) Secondary structure of JAK molecules. They are composed (from the N- to C-terminal end) of a FERM domain (band 4.1, erzin, radixin, moeisin domain), a pseudo-SH2 domain (ψ-SH2), a pseudokinase domain (JH2), and a kinase domain (JH1). While the major function of the N-terminus region is to specifically bind to cytokine receptor intracellular tail, the carboxyl-terminal region contains the catalytically active kinase domain, JH1, and the regulatory domain, JH2, of which the exact function is still matter of debate. ( **B**) Three-dimensional representation of JAK2 kinase domain in its active conformation (PDB: 3KRR ^12^) bound to type 1 inhibitor, ruxolitinib (show in yellow spheres). The binding of ruxolitinib has been modelled on the basis of the co-crystal of ruxolitinib-bound Scr (PDB: 4U5J ^13^). The activation loop, colored in pink, adopts an ‘open’ (active) conformation stabilized by phosphorylation of tyrosine residues 1007 and 1008. The αC is colored in orange. ( **C**) Three-dimensional representation of JAK2 kinase domain in its inactive conformation bound to a type 2 inhibitor, NVP-BTT594 (PDB: 3UGC ^14^). The activation loop, colored in pink, adopts a ‘closed’ (inactive) conformation. The compound is shown in cyan spheres, and the αC in orange. (D) Illustration of the structures of the main compounds discussed in this review.

The human genome codes for four JAKs: JAK1, JAK2, JAK3, and TYK2 ^1^. Like the god Janus, who has two faces and opens doors, JAKs possess two kinase domains—one catalytically active domain at the C-terminus and an upstream pseudokinase domain that binds ATP—but does not phosphorylate substrates (except weakly itself). At the N-terminus, JAKs possess a FERM (band four-point-one, ezrin, radixin, moesin)-like domain and an Src homology 2 (SH2)-like domain ( Figure 3A). The non-covalent attachment of JAKs to cytokine receptor tails is specific and depends on the FERM domain and on the juxtamembrane receptor sequence containing a proline-rich Box 1 and a motif denoted Box 2 with both hydrophobic and negatively charged residues as well as the sequences stretching from Box 1 to Box 2 ^6, 7^. JAK2 associates with the three homodimeric receptors: erythropoietin receptor (EPOR), thrombopoietin receptor (MPL/TPOR), and the granulocyte colony-stimulating factor receptor (G-CSFR/CSF3R). JAK1, JAK2, and TYK2 can also associate with heterodimeric or trimeric receptors, and JAK3 only with receptors containing the γ-chain with solely JAK1 as partner ( Figure 1) ^1^. The precise structure of receptor/JAK complexes remains unknown. Recently, the structures of the kinase and pseudokinase domains of JAK1, JAK2, and TYK2 were obtained and represent a major asset for the identification of novel (allosteric) inhibitors (see below) ^8– 10^. Interestingly, JAKs also play a role of chaperones for traffic and stability at the cell surface of several cytokine receptors ^11^.

## Disease associated with abnormal JAK activation

Autonomous activation of the JAK/STAT pathway is central in several pathologies. Genetic alterations targeting this signaling pathway are associated mainly with hematologic malignancies. Pathological JAK activation also occurs in diseases linked to abnormal cytokine stimulation.

### Hematologic malignancies

In many myeloid and lymphoid malignancies, driver mutations leading to constitutive JAK activation can be found. The paradigm is represented by *BCR-ABL1*–negative MPNs as they are all related to mutations activating JAK2, which in more than 50% of the cases both initiate and drive the disease phenotype. Thus, the development of JAK2 inhibitors is particularly critical for MPNs.

***Classic *BCR-ABL1*–negative MPNs.*** The classic *BCR-ABL1*–negative MPNs (hereafter, MPNs) include three different disorders—essential thrombocythemia (ET), polycythemia vera (PV), and primary myelofibrosis (PMF)—and are caused by constitutive activation of the cytokine receptor/JAK2 pathway due to acquired somatic mutations in three major genes ^15^. *JAK2*V617F is the most prevalent mutation in MPNs associated with the three disorders (65–70%) and is present in 95% of PVs. Mutations in the exon 12 of *JAK2* are found in around 2% of PV, which are negative for the *JAK2*V617F mutation (reviewed in 15). Interestingly, these two *JAK2*-activating mutations are not located in the kinase domain of the protein but involve amino acid changes in the pseudokinase (JH2) domain ( *JAK2*V617F) and the SH2-JH2 linker domain ( *JAK2 exon 12*) ( Figure 3A and see below). Mutations in the thrombopoietin receptor ( *MPL*) gene are much rarer than *JAK2* mutations and are present in 3% of MPNs (4–5% of ET and myelofibrosis [MF]). The recurrent mutations are located in the exon 10. The most frequent are substitutions of the W515 residue to many other amino acids, mostly L or K but also R or A (reviewed in 15). In MPNs, the *MPL*S505N mutation is also found but less frequently than in hereditary thrombocytosis. Rarer mutations of *MPL* have been described in both the extracellular and intracellular domains. The third gene found frequently mutated in MPNs is calreticulin ** ( *CALR*), which is affected by mutations leading to a +1 frameshift in the exon 9 ^16, 17^. As for *MPL*, *CALR* mutations are associated with ET and MF but with a higher frequency (25%). CALR is not a molecule directly involved in activation of JAK2, but the new C-terminus common to all mutants allows the CALR mutants to tightly bind and activate MPL and JAK2 ^18– 21^.

All of these activating mutations mimic the effects of hematopoietic cytokines by inducing constitutive signaling via the STAT, PI3K, and ERK/MAPK pathways. To achieve this, *JAK2*V617F must be bound to cytokine receptors, more particularly homodimeric receptors (EPOR, CSF3R/G-CSFR, and MPL/TPOR), which allow its dimerization and activation ^22^. CALR mutants specifically activate MPL and (to a lesser extent) G-CSFR, and *MPL* mutations result in an active conformation of the receptor ^18^. Loss-of-function mutations in *SH2B3*, a negative regulator of JAK2, have been described as drivers in MPNs and idiopathic erythrocytosis ^23, 24^, but there is evidence that in the majority of the cases it is either a secondary mutation or a germ-line mutation predisposing to MPNs ^25^.

In 20–30% of patients with MPNs, mutations besides driver JAK2-activating mutations have been identified in genes involved in splicing ( *SRSF2* and *U2AF1*) and epigenetic regulation ( *TET2*, *DNMT3A*, *ASXL1*, *EZH2*, and *IDH1/IDH2*) and in tumor suppressor genes ( *TP53*) ^15, 26, 27^. They could predate or follow the driver mutations ^28– 30^. Mutations, such as in *TET2*, *DNMT3A*, or *EZH2*, favor clonal dominance and disease initiation ^28, 31^. Of note, these associated mutations promote progression to MF or leukemic transformation ( *ASXL1*, *IDH1/2*, *EZH2*, and *TP53*) ^32, 33^. Almost always, one such mutation is present in PMF, and the number of such mutations correlates with the severity of the disease ^32, 33^ and might modulate the response to JAK2 inhibition.

***Other myeloid malignancies***

**MPN associated with JAK2 fusion proteins**

Four fusion proteins—ETV6 (TEL)-JAK2 (t(9;12) (p24;p13), PCM1-JAK2 (t(8;9) (p22;p24), BCR-JAK2 (t(9;22) (p24;q11.2), and RPN1-JAK2 (t(3;9) (q21;p24)—have been described in some MPNs—PMF, chronic eosinophilic leukemia (CEL), atypical chronic myeloid leukemia (aCML), and unclassified MPNs—or in mixed MPN/myelodysplastic syndrome (MPN/MDS) ^34– 37^. The translocation partner of JAK2 sets the expression level and induces dimerization of JAK2 kinase domains.

**Chronic neutrophilic leukemia, an MPN associated with G-CSFR (** *CSFR3* **) mutations**

Acquired activating *CSF3R* mutations have been found in the great majority of chronic neutrophilic leukemias (CNLs). Most CNLs carry a T618I mutation (T595I if the first counted residue is after the peptide signal sequence) ^38^. The T615N (T592N) mutation has also been described but more rarely. *CSF3R* mutations have also initially been described in aCML but are much less frequent than in CNL.

*JAK2*V617F and (to a lower extent) *MPL* and *CALR* mutations are extremely prevalent in refractory anemia with ring sideroblasts and thrombocytosis and are associated with *SF3B1* mutations ^39^.

Mutations in *JAK2* or cytokine receptors are rare in the other myeloid malignancies. They can be found in chronic myelomonocytic leukemia (CMML) but always associated with other mutations.

In acute myeloid leukemia (AML), mutations in *JAKs* are rare and, when present, mostly involve *JAK1*. *CSF3R* mutations have also been described but as late events. The only AML associated frequently with *JAK* mutations is Down syndrome acute megakaryoblastic leukemia (AMKL), where *JAK1*, *JAK2*, and also *JAK3* mutations are found in around 20% of cases ^40, 41^.

In acute myeloid leukemia (AML), mutations in *JAKs* are rare and, when present, mostly involve *JAK1*. *CSF3R* mutations have also been described but as late events. The only AML associated frequently with *JAK* mutations is Down syndrome acute megakaryoblastic leukemia (AMKL), where *JAK1*, *JAK2*, and also *JAK3* mutations are found in around 20% of cases ^40, 41^.

***Lymphoid malignancies.*** JAK2 activation may play a more significant role in the pathogenesis of B and T neoplasms than previously thought, although they might be only secondary events. JAK/STAT activation occurs via either mutations/translocations or cytokine paracrine/autocrine loops.

**Acute lymphoblastic leukemia**

In B-cell acute lymphoblastic leukemia (B-ALL), a new subtype has emerged called *BCR-ABL1*-like subtype (15% of pediatric ALL and 50% of ALL with Down syndrome), as it exhibits a transcriptional profile similar to that of *BCR-ABL1* **–**positive ALL with poor prognosis ^42^. Half of them have *JAK1* and *JAK2* mutations and rearrangement of *CRLF2*, a gene encoding a cytokine receptor ^43, 44^. The most frequent *JAK2* mutations target the R683 (R683G/S), a residue present in the DIREED motif located in the hinge between the N- and C-lobes of the pseudokinase domain of JAK2 ^44, 45^. Fusion with partners ETV6, BCR, PAX, and SSBP2 results in the activation of JAK2 kinase domain. Mutations in *JAK1* are relatively rare in B-ALL in comparison with T-cell ALL (T-ALL) ^46^ and are located in the FERM domain and the pseudokinase domain. For instance, *JAK1*V658F is the equivalent of *JAK2*V617F ^47^. In most cases, *JAK2* mutations are associated with an aberrant expression of CRLF2, a cytokine receptor chain which associates with IL-7RA to bind the thymic stromal lymphopoietin (TSLP) ^48^. The F232C-activating mutation in *CRLF2* induces homodimerization of CRLF2 and is detected in 10% of cases overexpressing CRLF2 ^49^. Activating mutations in the *IL-7RA* have been described either as point mutation (S185C) or as an insertion-deletion in the transmembrane domain inducing homodimerization of the IL-7RA ^50^. Truncating rearrangements of the *EPOR* have also been identified ^51^.

In T-ALL, mutations in *JAK1*, *JAK2*, *JAK3*, and the *IL-7R* are found in around 15% of cases, more particularly in early T-cell progenitor (ETP)-ALL ^52^. Targeting JAKs in these two types of ALL appears to be a valuable approach.

**Hodgkin lymphoma and primary mediastinal B-cell lymphoma**

Hodgkin lymphoma and primary mediastinal B-cell lymphoma (PMBL) have some common mechanisms of lymphomagenesis, being driven by alterations in the nuclear factor-kappa B (NFκB) and JAK/STAT pathways ^53, 54^. In rare cases (3%), a JAK2 fusion protein (SEC31A-JAK2) is present ^55^.

**Other lymphoma**

In T-cell pro-lymphocytic leukemia, mutations in *JAK1* (8%) and *JAK3* (30%) (more particularly M511I) have been described ^56^. *JAK3* mutations (particularly A572V and A573V) are frequent (35%) in natural killer/T-cell lymphomas ^57^. Mutations or silencing of negative regulators of JAKs ( *PTPN2* and *SOCS1*) is also frequent in diffuse large B-cell lymphoma, follicular lymphoma, and peripheral T-cell lymphoma.

An autocrine/paracrine cytokine loop induced by IL-6, IL-10, and IL-13 activates JAKs. This occurs in several types of lymphoma as well as in chronic lymphocytic leukemia and Waldenström macroglobulinemia ^58^.

### Disorders other than hematologic malignancies

***Inherited disorders of the hematopoietic system.*** The majority of inherited thrombocytosis are related to spontaneous activation of the MPL/JAK2 pathways due to *MPL* or *JAK2* mutations ^59, 60^. The other mechanism is related to an excess of plasma thrombopoietin (TPO) due either to an excessive synthesis or to a defect in its clearance as a consequence of *MPL* mutations affecting receptor trafficking. This excess of TPO induces JAK2 activation ^59, 60^.

Inherited erythrocytosis are related to excess erythropoietin (EPO) synthesis or to gain-of-function mutations in *EPOR* that activate JAK2 ^60^. Hereditary neutrophilia carry activating mutations of *CSF3R* that activate JAK2 ^61^.

***Inflammatory and autoimmune diseases.*** A large spectrum of diseases of the immune system involves an activation of JAKs through autocrine or paracrine cytokine loops ^62^. In inflammatory pathologies, such as rheumatoid arthritis (RA), psoriasis, inflammatory bowel disease, and alopecia areata, the most important JAK to be targeted is JAK1 ^62^. In autoimmune disease, JAK1 is also the main JAK to be targeted as both the IL-6 and the type 1 interferon (IFN) pathways are involved. Blocking JAK1/2 in graft-versus-host disease (GVHD) is useful as type II IFN and IL-6 are pathogenic ^63^. Lastly, JAK inhibition can be useful to curb oncogenic inflammatory responses in a wide range of solid tumors.

## JAK inhibitors

Several types of inhibitors exist according to their mechanism/region targeted in JAKs ^64^.

### Type I inhibitors

Type I inhibitors target the ATP-binding site of the JAKs under the active conformation of the kinase domain ( Figure 3B) ^64^. All clinically tested inhibitors are type I. They differ in their specificity for each JAK. Many inhibitors target JAK2, JAK1, and eventually TYK2 (ruxolitinib, momelotinib, AZD1480, and baricitinib) or JAK3 and JAK1 (tofacitinib). Some are pan-JAK inhibitors (gandotinib, XL019, NVP-BSK805, peficitinib, and pyridone 6). Less frequently, they target only JAK2 (NS-018, pacritinib, CEP-33779, NVP-BVB808, TG101209, fedratinib, and AZD960), JAK1 (filgotinib and itacitinib), or JAK3 (decernotinib, janex1, and JAK3-IN-1). However, they also target other kinases, in particular FLT3 (pacritinib, NVP-BVB808, TG101209, and fedratinib), Src (NS018), or Aurora A (AZD1480) ( Table 1 and Figure 3B).

**Table 1.** JAK inhibitors, their targets, and their applications to pathologies.

Inhibitors	Selectivity	Off- target	Diseases	Clinical phases
**Type I**				
Ruxolitinib	JAK2>JAK1>JAK3		MF and hydroxyurea resistant or intolerant PV Refractory leukemia (post-MPN leukemia) Pancreatic cancers Corticosteroid refractory-GVHD Psoriasis Alopecia Vitiligo	FDA-approved ^77– 79^ Phase 2 ^80^ Phase 2 in combination with capecitabine (after gemcitabine failure) ^81^ In evaluation ^82^ Phase 2 ^83^ Open-label clinical trial ^84^ Case report ^85^
Momelotinib (CYT-387)	JAK2>JAK1>JAK3	ALK-2 TBK1 IKKε	PMF Post PV/ET MF PV/ET	Phase 3 - SIMPLIFY-1/2 (Stopped) ^86^ Phase 2 (terminated) ^87^
AZD1480	JAK2>JAK1	Aurora A FGFR1 FLT4	PMF Post PV/ET MF B-ALL Solid tumors	Phase 1 (completed) ^88^ In evaluation (preclinic) ^89^ Phase 1 (terminated) ^90^
Baricitinib (INCB-028050)	JAK2>JAK1		Rheumatoid arthritis Psoriasis	Phase 3 (FDA approval in process, EMA-approved) ^91^ Phase 2 ^92^
Tofacitinib	JAK1>JAK3		Methothrexate-resistant rheumatoid arthritis Crohn’s Psoriasis Alopecia areata Dermatomyositis, vitiligo	FDA-approved ^93^ Phase 2 ^94^ Phase 3 ^95^ Phase 2 ^96^ Case report ^96^
Gandotinib (LY2784544)	Pan-JAK JAK2V617F>JAK2		JAK2V617F-positive MF, ET and PV patients	Phase 1 ^97^ Phase 2 (in progress)
XL019	Pan-JAK		PV, MF	Phase 1 (terminated) ^98^
NVP-BSK805	JAK2		JAK2V617F	Cellular models ^99^
NS-018	JAK2V617F>JAK2	Src	PMF, post PV/ET MF patients JAK2V617F selective	Phase 2 ^12^ In vitro ^100^
Pacritinib (SB11518)	JAK2	FLT3	MF	Phase 3 PERSIST-1 ^101^, PERSIST-2 ^102^ PAC203 study evaluating the effect of lower doses
CEP-33779	JAK2		Rheumatoid arthritis, colorectal cancer, lupus nephritis	Preclinical mouse models ^103^
NVP-BVB808	JAK2	FLT3	MPN	Cell lines ^104^
TG101209	JAK2	FLT3	MPN, systemic sclerosis	Cellular models ^105^
Fedratinib (TG101348)	JAK2	FLT3 BRD4	MF	Phase 3 JAKARTA ^106^ FDA removed the clinical hold in August 2017
AZ960	JAK2		ATL, other leukemia	Cell lines
Filgotinib (GLPG0634)	JAK1>JAK2		Rheumatoid arthritis Bowel and Crohn’s diseases Lupus and psoriasis	Phase 3 ^107^ Phase 2 ^108^ Phase 2 ^109^
Itacitinib (INCB-039110)	JAK1		MF Psoriasis Non-small cell lung cancer GVHD B-cell lymphoma BRAF-mutant melanoma and other solid tumors	Phase 2 (alone or in combination with low-dose of ruxolitinib) ^110^ Phase 2 ^111^ Phase 2 (combination with EGFR inhibitor, osimertinib) (in progress) Phase 3 (combination with corticosteroids) (in progress) Phase 1/2 (combination with BTK inhibitor, ibrutinib) (in progress) Phase 1 (in combination with MAPK inhibitors, dabrafenib or trametinib) (in progress)
INCB52793	JAK1		Advanced malignancies	Phase 1 (in progress)
PF-04965842	JAK1		Moderate to severe psoriasis	Phase 2 ^112^
Upadacitinib (ABT-494)	JAK1		Rheumatoid arthritis	Phase 2 ^113^ Phase 3 (in progress)
Decernotinib (VX-509)	JAK3		Rheumatoid arthritis	Phase 2/3 ^114^
WHI-P131/ JANEX-1	JAK3		GVHD	Preclinical mouse model ^115^
JAK3-IN-1	JAK3		N/A	N/A
Peficitinib (ASP015K)	JAK3		Psoriasis Rheumatoid arthritis	Phase 2 ^116^ Phase 2 ^117^
**Type II**				
NVP-BBT594	JAK2	BCR-ABL KDR FLT3 RET		Cellular models ^14^
NVP-CHZ868	JAK2	KIT, PDGFR VEGFR	MPN B-ALL	Preclinical mouse models ^75^
**Allosteric** **inhibitors**				
LS104	JAK2	BCR- ABL	MPN	JAK2V617F cell lines ^118^
ON044580	JAK2	BCR- ABL	MPN	BCR-ABL cell lines ^119^

ATL, adult T-cell leukemia; B-ALL, B-cell acute lymphoblastic leukemia; BTK, Bruton’s tyrosine kinase; EGFR, epidermal growth factor receptor; EMA, European Medicines Agency; ET, essential thrombocythemia; FDA, US Food and Drug Administration; GVHD, graft-versus-host disease; JAK, Janus kinase; MAPK, mitogen-activated protein kinase; MF, myelofibrosis; MPN, myeloproliferative neoplasm; N/A, not applicable; PMF, primary myelofibrosis; PV, polycythemia vera.

The differences in specificities for JAK are the basis for the different trials: JAK2 specificity for MPNs and certain malignant disorders ^65, 66^ and JAK1 and JAK3 for inflammation and auto-immune diseases ^67^. The clinical toxicity can be related to the precise JAK protein that is inhibited—hematological toxicity, eventually immune suppression for JAK2 ^66^,—immune suppression and long-term effects on hematopoietic stem cells (HSCs) for JAK1 and JAK3 ^68^. It can be also due to an off-target inhibition. It has been suggested that the gastrointestinal toxicity was related to FLT3 inhibition ^69^ and the Wernicke encephalitis observed in rare patients treated with fedratinib to inhibition of thiamine uptake ^70^.

Currently, only three JAK inhibitors are US Food and Drug Administration (FDA) or European Medicines Agency (EMA)-approved for treatment: ruxolitinib for the treatment of MF and hydroxyurea (HU)-resistant or -intolerant PVs ^65^, tofacitinib and Baricitinib for the treatment of methotrexate-resistant RA ^71^.

The major limitation of type I inhibitors that bind to active state kinases is that while they block catalysis they allow increased phosphorylation of the activation loop on Y1007, which upon overexpression of JAK2 or other JAKs can create heteromeric JAK complexes that re-set signaling ^72^. This might explain why resistance to JAK2 inhibition is not related to mutations but to functional inhibition ^73^.

### Type II inhibitors

The type II inhibitors bind to the ATP-binding pocket of kinase domains in inactive conformation, and the F of the DFG pocket is in an out conformation ( Figure 3C) ^64, 74^. Inhibition is more efficient and is not reversed by drug detachment ^14^. The best example is imatinib and the second generation of BCR-ABL inhibitors ^74^. Two type II JAK2 inhibitors (NVP-BBT594 and NVP-CHZ868) have been developed. NVP-CHZ868 has been used in preclinical models and was very effective ^75, 76^. Both inhibitors were not amenable for drug development. Owing to their powerful activity, the type II JAK2 inhibitors present the risk of inducing profound cytopenia, limiting its future use in PV or ET.

### Allosteric inhibitors

Allosteric inhibitors are molecules that do not bind to the active kinase site but to another site ^64^. Theoretically, they would be more specific than an ATP-pocket inhibitor given the high homology of ATP-binding sites. Allosteric inhibition could be interesting to specifically target only the mutated JAKs ( *JAK2*V617F: 60–70% of MPNs and JAK2 exon 12: 1%) as JAK2WT is indispensable for normal hematopoiesis. An efficient JAK2WT inhibition will always lead to a cytopenia.

Within allosteric inhibitors, type III bind to a site close to the ATP-binding site while type IV bind to an allosteric site distant from the ATP-binding site. This means that in the case of *JAK2*V617F it might be important to target the pseudokinase domain (type IV inhibitors, see last section). To our knowledge, there is currently no allosteric inhibitor of *JAK2*V617F in development.

### Other types of inhibitors

For kinases other than JAKs, additional types of inhibitors have been developed ^64^:

Type V. Such inhibitors reversibly bind to two sites of the kinase domain.

Covalent. They irreversibly bind on a nucleophilic residue (usually a cysteine) located in the ATP-binding site.

## JAK inhibitor therapies

### *BCR-ABL* –negative MPNs

Triggered by the discovery of *JAK2*V617F, JAK2 inhibitors have been developed, but none of them is specific to the mutant protein and most of them also target other kinases such as JAK1 and FLT3. One advantage is that their use can be extended to the *JAK2*V617F-negative MPNs or other pathologies associated with JAK2 activation that include inflammatory diseases and certain other cancers. Theoretically, it is not conceivable to completely inhibit JAK2 in the long term, because this will lead to a profound cytopenia and eventually aplastic anemia. Thus, these inhibitors can be used because they only partially inhibit JAK2 *in vivo* for different reasons (pharmacokinetics and resistance). This explains why such inhibitors give similar clinical results in MF’s improving quality of life, decreasing the splenomegaly, and improving survival. The evolution of the disease is not changed, nor apparently is the rate of MPN transformation. The differences between inhibitors concern the side effects that may essentially be related to distinct off-targets ( Table 1).

***Ruxolitinib.*** Ruxolitinib ( Figure 3B and D) was the first JAK2 inhibitor approved for therapy of MF (high and intermediate risks). It is now the only JAK inhibitor for which a long-term follow-up has been reached. In intermediate-2 and high-risk MF, a very significant effect is found in more than 50% of the patients with a reduced spleen size (clinical trials COMFORT-1 and COMFORT-2) at any point in the trial and even a more marked effect on the general symptoms, in particular the pruritus ^77– 79^. These results have been extended to low- and intermediate-1 risk MF ^120^. In HU-refractory PVs, ruxolitinib allows the control of hematocrit in more than 60% of patients and induces a spleen volume reduction in 38% of cases at 32 weeks. In addition, a molecular decrease of *JAK2*V617F allele burden reaching a mean of 40% at 208 weeks was observed ^78, 121^. In HU-resistant or -intolerant ET, there are divergent results. In one study, ruxolitinib offered no advantage compared with other therapies in the control of the thrombocytosis and disease complications but did alleviate general symptoms and pruritus ^122^. In the other ^123^, which was an open-label phase 2 trial, ruxolitinib induced a meaningful reduction in platelet levels and attenuated ET-related symptoms. These preliminary results seemed superior to historically observed results, but this study was done in the absence of a comparison with another treatment.

Overall, ruxolitinib is a well-tolerated oral treatment with approximately 25–33% of adverse effects. The main toxicities are hematological, moderate anemia that may correct with time, and thrombocytopenia, which can be very severe in high-risk MF. Weight gain is also observed with possible abnormalities in lipid metabolism. Middle-term toxicity is an immune suppression that may be responsible for reactivation of viral infections, particularly herpes zoster and HIV1 and bacterial infections such as pneumonia, tuberculosis reactivation and urinary tract infections ^124, 125^. Long-term monitoring will be important because ruxolitinib decreases natural killer cell functions with a potential risk of solid tumor and lymphoma development ^126, 127^. This is particularly important if indications are extended to low-risk MF, PV, and ET. Analysis of patients treated for several years with ruxolitinb indicates an increase in survival in MF, but progression to leukemia is not significantly different ^128^. It is possible that most pro-survival effects derive from its palliative anti-inflammatory effects ^65^.

***Other JAK2 inhibitors.*** Momelotinib (CYT38) ( Figure 3D) is a JAK1/JAK2 inhibitor that has shown activity resembling ruxolitinib with respect to spleen size reduction and constitutional symptom alleviation ^129– 131^. Importantly, momelotinib was shown to ameliorate anemia, which is a major concern in MF. The mechanism appears to be the reduction of hepcidin production through a direct inhibition of the activin receptor-like kinase-2 (ALK-2) ^132^. Thus, momelotinib was thought to be an alternative to ruxolitinib for patients with anemia. These promising results led to the opening of a phase III trial for the SIMPLIFY-1 and -2 studies in MF. However, the results of the two clinical trials did not show a major advantage of momelotinib on ruxolitinib, although momelotinib was associated with a decrease in transfusion requirement ^86^. Momelotinib development has been stopped.

NS-018 ( Figure 3D) is a JAK2/Src inhibitor that has been assessed in patients with *JAK2*V617F-positive MF, ET, and PV. NS-018 shows an apparent increased potency for the JAK2V617F mutant in mouse models, possibly leading to less immunosuppressive effects ^100^. It was tested in MF with symptom improvement but minor impact on the numbers of *JAK2*V617F cells ^133^. Gandotinib (LY2784544) ( Figure 3D) is a potent JAK2 inhibitor, which also exhibits a certain selectivity toward JAK2V617F. It was evaluated for safety and tolerability in ET, PV, and MF ^97^.

Pacritinib (SB1518) ( Figure 3D) is a JAK2/FLT3 inhibitor. Promising results were obtained in phase 1–2 clinical trials. It showed good activity in patients with less immunosuppressive effects ^134^. Pacritinib could be administered to patients with low platelet levels, as it does not induce thrombocytopenia. The reasons behind this feature are unclear; they could be linked to reduced specificity for MPL/JAK2 complexes. Subsequently, two phase 3 clinical trials (PERSIST 1 and 2) were started with different doses of pacritinib. In 2016, the FDA put a complete clinical hold on the trials because of an excess of intracranial hemorrhage and cardiac failure in treated patients ^135^. This hold was lifted in 2017 ^136^. As a consequence, CTI BioPharma has just launched the PAC203 study evaluating the effect of pacritinib at different doses.

Fedratinib (TG101348) ( Figure 3D) was assessed during the JAKARTA trials with interesting clinical results, including fibrosis reduction ^137– 139^, but rare patients developed Wernicke encephalopathy, which led to its stop ^139^. It was assumed that it was related to an inhibition of thiamine uptake, although fedratinib does not lead to inhibition of thiamine uptake in rats ^70, 140^. FDA removed the clinical hold in august 2017 and clinical trials are being planned in 2018.

JAK1 inhibition has also been proposed in MPNs, as an anti-inflammatory strategy, and an alternative to JAK2 inhibitors to avoid anemia and thrombocytopenia ^65, 66^. A preliminary study has produced mixed results with a modest reduction in spleen size ^110^. Recently, it has been demonstrated that JAK1 plays essential and non-redundant roles at the level of HSC and therefore long-term JAK1 inhibition might have negative effects on HSCs ^68^.

### Other malignant disorders

Ruxolitinib was also used in refractory leukemia, including post-MPN leukemia, and 3 out of 18 patients achieved complete remission ^80^. Ruxolitinib was unexpectedly used in combination with chemotherapeutic agents in solid cancers (pancreatic and lung cancer) and there was some benefit ^141^. This was based on JAK1 involvement in the signaling of several cytokine receptors, inflammation, and possibly progression of the malignant clone ^81, 142^.

Ruxolitinib has been used both in myeloid malignancies with JAK2 fusion proteins and in CNL with a very good initial response but was inefficient to cure the diseases ^143– 146^.

Itacitinib (INCB39110), which is also a selective JAK1 inhibitor, is being tested in non-small cell lung cancer in combination with an epidermal growth factor receptor inhibitor. Another JAK1 inhibitor, INCB52793, is also in development in advanced malignancies.

### Inflammatory and autoimmune diseases

JAK1 inhibitors have been used in inflammatory/immune diseases ^67, 147^. Tofacitinib was authorized by the FDA in RA ^148^ but is currently being tested in Crohn’s disease, psoriasis, and other diseases ^149, 150^. It decreases inflammation particularly by lowering T-cell and macrophage infiltrates. Filgotinib is currently in phase 3 clinical trials in RA and Crohn’s disease and in phase 2 clinical trials in lupus and psoriasis. Itacitinib (INCB39110) is being tested in phase 2 trials in psoriasis and in MF with meaningful improvements.

Ruxolitinib, also a good inhibitor of JAK1, was shown to reduce GVHD in mice and in patients with corticosteroid-refractory GVHD ^82, 151^. Indeed, in GVHD, severe complications are due to high levels of proinflammatory cytokines that are inhibited by ruxolitinib. Itacitinib will be assessed as monotherapy in GVHD.

A JAK1 inhibitor, PF-04965842, is also in clinical trials in atopic dermatis and severe psoriasis ^112^. Both topical tofacitinib and ruxolitinib are tested for alopecia and vitiligo.

Baricitinib (Olumiant), a JAK1/2 inhibitor (half maximal inhibitory concentration [IC _50_] values of 5.9 and 5.7 nM, respectively), has been initially identified by Incyte and subsequently developed by Eli Lilly and Company for RA. It also inhibits TYK2 (IC _50_ of around 53 nM). A phase 3 clinical trial was conducted, and the molecule was approved by the EMA but not yet by the FDA ^148, 152^. It may be of interest in MPN treatment given its similarities with ruxolitinib but with a longer half-life (12.5 hours). Upadacitinib (ABT-494, AbbVie) is a highly selective JAK1 inhibitor that will enter phase 3 trials for RA, psoriasis, and ulcerative colitis.

Two JAK3 inhibitors were evaluated. ASP015K, also designated JNJ-54781532, displays a moderate selectivity on JAK3 over JAK1 and JAK2. It was shown to induce efficacy and safety in psoriasis ^116^. It has demonstrated efficacy in preclinical models of RA and dermatitis. Decernolitinib is also a potent and selective inhibitor of JAK3 developed as a second-generation inhibitor in autoimmune diseases, particularly in RA. However, at high doses, it leads to anemia, indicating that its selectivity *in vivo* could be different ^153^. ** In MPNs, these types of inhibitors could also be useful to decrease inflammation, especially in MF.

The major drawback of these JAK1 and JAK3 inhibitors affecting the inflammatory response is that they can induce autoimmune diseases (thyroiditis or myocarditis) or can prime the development of many infections in patients with MPN. Furthermore, it appears that for the IL-2/IL-4/IL-7/IL-9/IL-15/IL-21 complexes, JAK1 is the initiating kinase for signaling, and a JAK3 that is inhibited can still fulfill its scaffolding role in the complex, its inhibition not giving results comparable to the absence of JAK3 by autosomal mutation ^154^.

## Perspectives in JAK inhibition

The goal of JAK2 inhibition in MPNs has switched from a curative therapy to a symptomatic and anti-inflammatory therapy with certain clinical benefits ^65^. However, this aim is far from what is expected for a targeted therapy ^69^. Although several *in vitro* studies identified potential kinase domain mutations that would give resistance to JAK2 inhibitors ^155, 156^, no such mutations were identified in patients ^72^, further arguing that the current inhibitors are weak. Thus, there is a need for new inhibitors or combination of therapies.

### New inhibitors

First, a new generation of JAK2-specific compounds, including allosteric inhibitors targeting unique sequences in JAK2, should come to light. Such inhibitors would provide more specificity toward JAK2 or JAK2V617F, thereby ameliorating normal JAK2 inhibition-based immune suppression. Two inhibitors have been developed (LS104 and ON044580) that inhibit kinase activities in a non-ATP-competitive manner ^118, 119^. LS104 preferentially inhibits JAK2V617F kinase and can synergize with ATP-competitive inhibitors ^118^, whereas ON044580 inhibits BCR-ABL and its T315I mutant ^119^. The precise targeted residues remain unknown.

Identification of allosteric sites in enzymes has been accomplished for several years and is the basis for the development of a new class of pharmaceuticals. One example is the development of the BCR-ABL allosteric inhibitor GNF-2 that can overcome the effect of resistant mutations and also exhibits an increased potency when used in combination with classic ATP-competitive inhibitors ^157^. Another example is the development of MEK allosteric inhibitors. It was shown that analogues of PD184352 could specifically bind to a unique region adjacent to the ATP pocket created by the displacement of the helix αC of the kinase in the active conformation ^158^.

**Figure 4.**
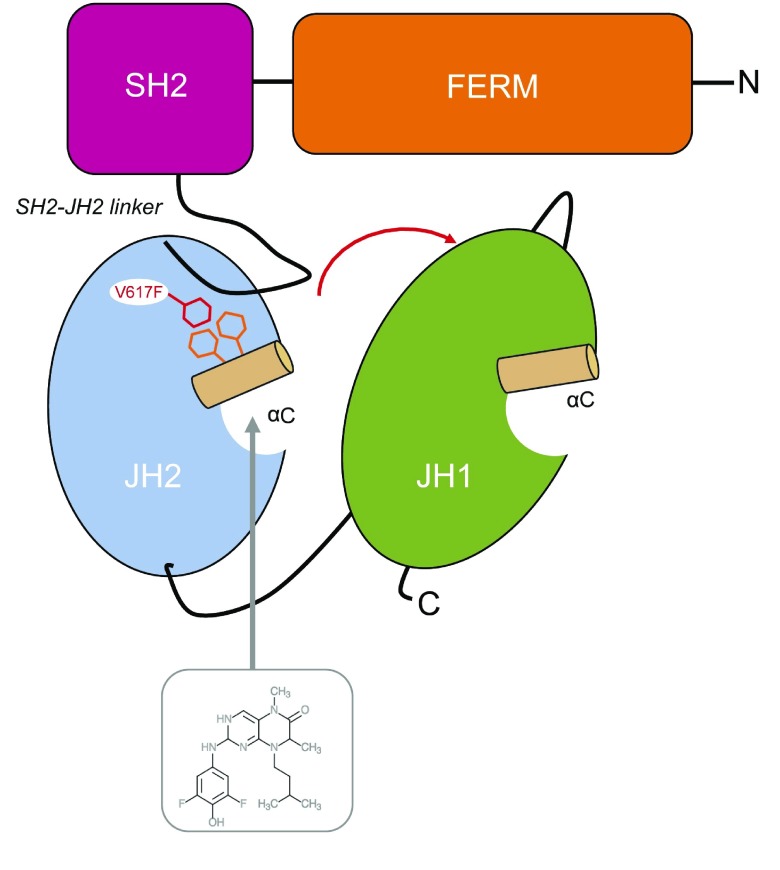
Graphic representation of the V617F-activation mechanism in Janus kinase2 (JAK2). The V617F activation has been suggested to derive from a combination of several molecular events triggered from a region surrounding the JH2 αC but also involving the SH2-JH2 linker. Phenylalanine 617 interacts with F594 and F595 from the JH2 αC and F537 from the SH2-JH2 linker, as supported by structural data ^9, 159^, then induces putative conformational changes that are transmitted to the adjacent catalytic kinase domain where activation is initiated. Targeting the ATP-binding pocket of JH2 that is spatially close the αC represents an appealing approach for specific targeting of the mutant JAK2V617F. Small molecules, such as BI-D1870 (as represented here ^160^), have been co-crystallized as bound to the JH2 ATP-binding site. The use of amendable compounds targeting the JH2 pocket has recently become a tantalizing concept and will represent the future challenge for drug design.

As the main conformational difference between JAK2V617F and JAK2WT is around the helix αC of the pseudokinase JH2 domain ^159^, a rational design of similar molecules targeting this region could be efficient ( Figure 4). Recent structural and mechanistic data on JH2 V617F might help in designing such small molecules. Moreover, targeting JH2 might become feasible as shown by the recent publication of co-crystals of JH2 with several compounds. Future therapeutics should target small conformational changes that are specific for a particular mutant protein or its constitutive activity.

The activating mutation, *JAK2*V617F, is located in the pseudokinase domain of JAK2 and allosterically regulates the activity of JAK2 kinase domain. Another strategy would be to interrupt these specific intramolecular changes induced by V617F on the kinase domain of JAK2. The aromatic phenylalanine at position 617 interacts with the αC helix phenylalanines 594 and 595 ^161^ and with phenylalanine 537 from the SH2-JH2 linker ^9^ to produce an autonomous activation to the adjacent catalytically active domain, JH1 ( Figure 4). Both of these aberrant hydrophobic contacts induce critical conformational changes that can be visualized on the JH2 V617F crystal structures ^9, 159^. Any small molecules that would interfere with these specific atomic configurations exclusively present in the conformation of JAK2V617F would most likely generate mutant-specific inhibitors. Evidence was provided that V617F activates JAK2 through a community of residues across JAK2 (from JH2 to JH1 through the SH2-JH2 linker) and that reversing the charge of the negative residue, E596, located on the solvent-exposed face of the JH2 αC helix, can efficiently uncouple JAK2V617F activation from cytokine-induced activation, thereby restoring auto-inhibition of JAK2.

Finally, methotrexate, a well-known drug used in autoimmune disease was shown to also inhibit the JAK/STAT pathway and in theory could be tested for selective effects on the MPN clones ^162^. An advantage of this treatment is its low cost, while its side effects are well known.

### Combination of therapies

The goal of certain combinations is to improve the anemia of MF, which can be worsened by ruxolitinib ^65^. Trials have been performed in combination with androgen without benefit. Trials are ongoing with pomalidomide or thalidomide and also with sotartercept, an activin receptor IIa ligand trap ^163^. Treatment with erythropoiesis-stimulating agents is also conceivable, although they are theoretically antagonistic with JAK2 inhibitors ^164^.

Downstream JAK2 are activations of STATs, PI3K-AKT/mTOR, and RAS-MAPK ERK1/2 ( Figure 2) ^165^. Specific inhibitors of STAT5 and STAT3 are now being tested in preclinical studies ^166^. A combination of JAK2 and STAT5 inhibitors might be effective in MPNs. In preclinical models, JAK2 and pan type I PI3K and mTOR inhibitors synergize to block JAK2V617F-induced proliferation ^167, 168^ but less on mutant CALR-induced proliferation ^18^. Other molecules involved in the inhibition of the PI3K, AKT, or mTOR pathway were tested in preclinical models or clinical trials.

The HSP90 chaperone is involved in JAK2 stability and is controlled via its acetylation status ^169^. HSP90 inhibitors or histone deacetylase inhibitors were tested ^169^. Notably, panobinostat and pracinostat were studied in combination with ruxolitinib in a phase 2 trial with a better spleen response in MF ^170^. Furthermore, a preliminary trial using an HSP90 inhibitor showed some clinical benefit ^171^. Combinations of ruxolitinib with CDK4/6 inhibitors, PIM1 kinase inhibitors, BH3 mimetics, or MDM2 inhibitors appear logical because they target molecules, which are downstream of JAK2 signaling, thus curbing any residual activation due to incomplete JAK2 inhibition due to short half-life of inhibitor and type I inhibition mechanism that allows rapid reactivation. Intriguingly, it has been reported that an association of ruxolitinib with pegylated interferon alpha was synergistic in a preliminary clinical trial, and a new larger clinical trial is ongoing ^172^. Normally, a JAK1 inhibitor should decrease interferon alpha signaling and should limit the effects of pegylated interferon; thus, the synergy might reflect how inefficiently ruxolitinib can actually inhibit JAKs *in vivo*. The main risk of all these different associations is to induce important cytopenia.

In MF, it has also been suggested to combine ruxolitinib with MEK inhibitors and PRM-151, a molecule that inhibits differentiation of fibrocytes ^173^, which are implicated in fibrosis development ^174^. Both drugs exhibit an effect on bone marrow fibrosis in preclinical studies or in clinical trials ^175^. An inhibitor of hedgehog signaling pathway (LDE225) was also tested in phase I in ET/PV and MF with disappointing results ^176^. However, recent evidence points to targeting Gli1 in association with ruxolitinib because Gli1 ^+^ mesenchymal cells may play a central role in fibrosis development ^177^.

JAK2V617F can increase the protein methylation demonstrated by activation of PRMT5 arginine methyltransferase ^178^. It also prevents the binding of heterochromatin factor HP1 to chromatin ^179^. It is possible that some key genes could be hypermethylated as in many cancers. Therefore, it was postulated that demethylating agents such azacytidine and decitabine may have some impact. However, administered alone, they showed minor responses in two clinical trials ^180, 181^. They were thus combined with ruxolitinib in a few patients with MF and the clinical response was good.

## Conclusions

After the discovery of *JAK2*V617F and the demonstration that *BCR-ABL*–negative MPNs are driven by abnormal JAK2 activation, there were curative expectations for JAK inhibitors. Despite nearly 10 years of development in MPNs, only one JAK2 inhibitor (ruxolitinib) has been clinically approved. Most other inhibitors had their development stopped because of neurotoxicity or the absence of superiority compared with ruxolitinib. Only pacritinib ( Figure 3B) is still in phase 3 clinical testing and fedratinib is being reevaluated. Although ruxolitinib offers clear benefits for patients, its effects are quite limited on the disease itself in MF. On one hand, this may be explained by the additional mutations detected by MF clones, which act independently from JAK2 and which place MF at the boundary between MPN and MDS. On the other hand, ruxolitinib has a short half-life and acts as a type I inhibitor, which means that each time inhibitor is consumed, re-activation of JAK2 will occur.

Thus, it seems important in PV and ET, which are JAK2-dependent MPNs, to obtain specific inhibitors of JAK2V617F or even to preferentially target the constitutive active JAK2 over cytokine-activated JAK2 in the cases of mutated *MPL* and *CALR* ET and MF. It is expected that such inhibitors will be less toxic and will really target the clonal disease. In MF, the low-hanging fruit of combination therapies could be a valuable approach that holds a risk for significant toxicities.

An important finding of the clinical trials in MPNs was the discovery that JAK inhibition is a valuable approach for treatment of inflammatory diseases. Thus, it can be expected that one of the main applications of JAK inhibitors will be for inflammatory diseases, autoimmune diseases, and possibly other diseases, which may include an inflammatory response, including neurodegenerative disorders or cancers where inflammation contributes to oncogenesis.
